# A deconvolution approach to modelling surges in COVID-19 cases and deaths

**DOI:** 10.1038/s41598-023-29198-4

**Published:** 2023-02-09

**Authors:** Adam Melnyk, Lena Kozarov, Sebastian Wachsmann-Hogiu

**Affiliations:** Department of Bioengineering, McGill University, 3480 Rue University, Montreal, QC H3A 0E9 Canada

**Keywords:** Medical research, Epidemiology

## Abstract

The COVID-19 pandemic continues to emphasize the importance of epidemiological modelling in guiding timely and systematic responses to public health threats. Nonetheless, the predictive qualities of these models remain limited by their underlying assumptions of the factors and determinants shaping national and regional disease landscapes. Here, we introduce epidemiological feature detection, a novel latent variable mixture modelling approach to extracting and parameterizing distinct and localized features of real-world trends in daily COVID-19 cases and deaths. In this approach, we combine methods of peak deconvolution that are commonly used in spectroscopy with the susceptible-infected-recovered-deceased model of disease transmission. We analyze the second wave of the COVID-19 pandemic in Israel, Canada, and Germany and find that the lag time between reported cases and deaths, which we term case-death latency, is closely correlated with adjusted case fatality rates across these countries. Our findings illustrate the spatiotemporal variability of both these disease metrics within and between different disease landscapes. They also highlight the complex relationship between case-death latency, adjusted case fatality rate, and COVID-19 management across various degrees of decentralized governments and administrative structures, which provides a retrospective framework for responding to future pandemics and disease outbreaks.

## Introduction

The COVID-19 pandemic has highlighted the importance of epidemiological modelling in responding to public health threats and continues to be a critical tool to study and anticipate the spread of disease, even with the introduction of vaccines and antiviral therapies. While there is an abundance of peer-reviewed research which sets out to make predictions about the short and long term spread of COVID-19 based on accepted epidemiological models, the study of past and emerging waves of the pandemic remains relatively unexplored despite the potential for understanding the impact of implemented public health responses. Some studies discuss the potential short term effects of nonpharmaceutical interventions such as mask mandates or lockdowns^1,2^ while others look deeper into possible disease futures, speculating about the longer term impacts of emerging variants, vaccination efficacies, and imperfect or waning immunities^3^. While these efforts broadly contribute to the decision making of public health authorities^4^, they often place an emphasis on forecasting over retrospective investigations that evaluate the accuracy of predictions^5^. Models of prediction are an integral part of the epidemiologist’s toolbox and serve as a basis for pandemic scenario planning; however, precise quantitative forecasting remains an imperfect assessment of future disease landscapes and public health risks^6,7^.

Deterministic compartmental models of disease transmission are among the most common modelling techniques in epidemiology. In these models, individuals are labeled and compartmentalized based on their disease status (e.g., susceptible, infected, recovered) and set to move between compartments over time according to model parameters and dynamics representative of a specified epidemiological landscape. Mean-field compartmental models assume that labeled populations are sufficiently large and homogenously mixed, such that variations in individual behaviours are approximated by a single averaged effect across an entire population^8^. Averaged analytical solutions of compartmental dynamics are generated from point estimates of model parameters, which provide simple approximations of disease progression but limit the ability to quantify model uncertainty, especially for long term forecasting^8,9^. While the simplifying assumptions of these models pose limitations on their predicative abilities, they provide a parsimonious framework for measuring and monitoring past and real-time trends in disease landscapes based on spatiotemporal data^2^.

To complement ongoing scenario planning initiatives for pandemic preparedness, science advisors and policymakers need to take an empirical approach to modelling disease landscapes. Empirical models are not intended to derive projections of disease progression but instead use analytical methods to interpolate and better estimate drivers of disease over time. These approaches to disease spread use epidemiological frameworks, such as compartmental models, to quantify and simulate real-world data as opposed to forecasting based on assumptions of epidemiological parameters such as reproduction number, contact rate, or critical vaccination threshold. They are often used to estimate such parameters^10^ and may also be used to assess the validity of projections set forth by predictive models^5,11^.

While compartmental models serve as a basis for studying disease landscapes, the ability of these models to provide spatiotemporal information on disease progression is limited by the granularity of the data being studied and by their capacity to extract and relate latent epidemiological features across different data types (e.g., cases and deaths). Motivated by the concept of feature detection used in the field of computer vision^12^, a feature represents a distinct and localized grouping of new cases, deaths, and other epidemiological data (e.g., hospitalizations) from a larger disease landscape. Latent (or hidden) features could include city-wide outbreaks that become indiscernible at a national scale or local superspreader events smoothed over amidst provincial data, and they often manifest asynchronously across cases and deaths to shape disease landscapes. On a broader scale, these features are often the result of many confounding real-world events which share the same spatiotemporal localizations.

Epidemiologists should have access to other mathematical tools and techniques to extract hidden features from disease landscapes and improve existing epidemiological frameworks where richer data inputs may be unavailable. Recently, functional principal component analysis was used to model time series COVID-19 data in France and quantify the positive effects of vaccine rollouts across the country’s local administrative regions^13^. In another study, spectral analysis methods were used to identify peaks in the frequency and periodicity of similar time series data from seven of the countries affected most by COVID-19 to highlight common weekly patterns unique to COVID-19 data reporting^14^. These approaches prove to be powerful tools for systematic retrospective studies of epidemiological time series data.

Retrospective epidemiological studies could also benefit from more granular analyses of disease progression by using tools designed to decompose time series data into temporally localized features. Here, we explore peak deconvolution, which describes the process of deconstructing overlapping data features into component peaks to extract hidden information about underlying phenomena. For example, in surface-enhanced Raman scattering (SERS), deconvolution of complex SERS spectra is used for the detection and characterization of molecular species based on the position and intensity of extracted peaks^15^. In an epidemiological context, similar peak deconvolution methodologies could be useful for the extraction of hidden features from disease landscapes to provide a previously unexplored perspective into the complex dynamics of the COVID-19 pandemic.

One epidemiological parameter which has remained central to calibrating and fitting pandemic models of disease progression is the apparent temporal lag between publicly reported daily COVID-19 cases and deaths, a phenomenon which we term “case-death latency” (CDL). CDL is in part determined by the inherent dynamics of the SARS-CoV-2 virus^16^ as well as by the unique physiological responses it imposes on each infected individual^17^. However, it is also influenced by other factors such as the quality of public health infrastructure, and centralized disease reporting and management^18,19^. While this latency is reported in the literature^20^ and estimated to range on average between 13 and 16 days from the onset of COVID-19 symptoms^21,22^, little has been done to quantify variations in CDL over time or consider these variations within a broader epidemiological framework. Oversimplification of CDL can misinform calculations of important epidemiological metrics such as case fatality rate (CFR), which is currently calculated based on crude estimates of CDL, if any^23^. Given that it is influenced by many confounding—and often unknown—variables and used to estimate important epidemiological metrics, CDL serves as a rich and complex metaparameter which implicitly codes for a variety of factors and determinants of disease. Decoding CDL could provide new insights into COVID-19 disease landscapes.

Here, we introduce a peak deconvolution method to deconstruct previous waves of daily case-death trends into smaller sub-waves. We use these sub-waves to isolate latent features of disease landscapes as well as track distinct and localized changes in disease progression over time. We also present an implementation of the susceptible-infected-recovered-deceased (SIRD) model to empirically simulate peak fits taken from CDL analyses. We show how this parsimonious model can be used to quantify feature parameters of deconvolved sub-waves to better understand drivers of disease progression such as rates of infection, death, and recovery. We analyze the second waves of the COVID-19 pandemic in Israel, Canada, and Germany, which are well-suited to showcase this method of epidemiological modelling as they encompass the only pre-vaccine responses to the pandemic in which centralized testing was widely available to these populations.

In this paper, we aim to demonstrate how peak deconvolution can be used in combination with the SIRD model to provide a novel method of quantifying key epidemiological feature parameters, such as CDL and CFR, with greater temporal resolution. This approach of epidemiological feature detection is intended to help to study and understand surges in COVID-19 as non-homogenous events which are the sum of many smaller latent contributions. With this approach, each component pair of case-death peaks can be attributed to specific biological, behavioural, and social—among other—factors and determinants of disease. Finally, we discuss how these findings can inform researchers and policymakers with actionable insights regarding: the outcomes of public health policies and pandemic responses being implemented across various governments and administrative structures; the effectiveness of COVID-19 testing programs and intensive care infrastructure; as well as the spread of COVID-19 variants, among other epidemiological considerations.

## Methods

### Data selection and processing

**Figure 1:**
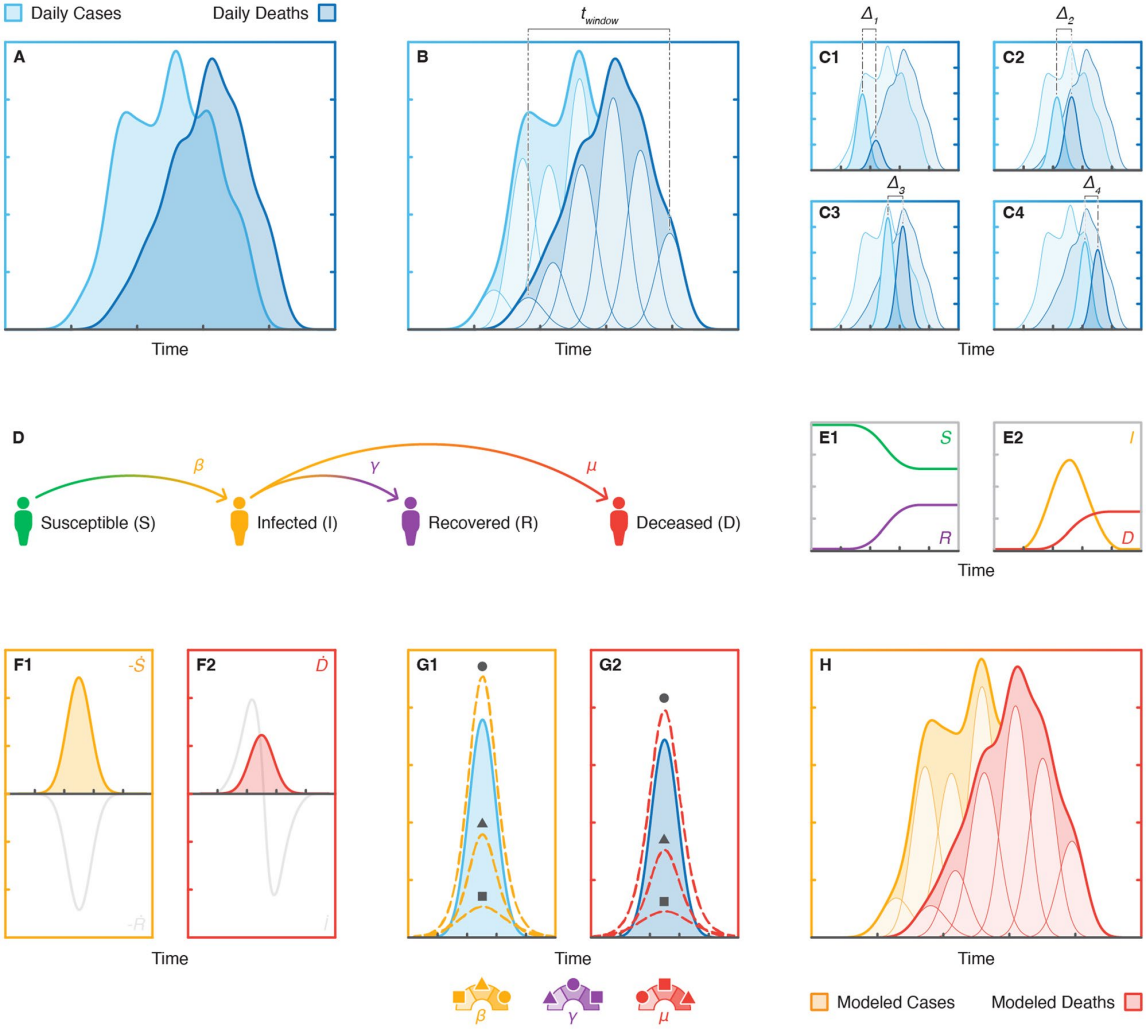
Epidemiological feature detection methodology: (**A**) reported daily cases and deaths data for a region of interest are cleaned and smoothed, (**B**) case and death trends are deconvolved into component peaks, (**C**) peak pairs are isolated for SIRD modelling, (**D**) a SIRD model is defined for each peak pair, (**E**) analytical solutions of the SIRD model are generated based on initial parameter inputs, (**F**) time derivatives of the model solutions are isolated to model peak pairs, (**G**) SIRD model parameters are optimized to fit each peak pair, (**H**) case and death trends are reconstructed using modelled peak pairs.

The COVID-19 case and death data used in this paper was downloaded from the Johns Hopkins Coronavirus Resource Center’s open access database^24^ and subsequently analyzed using our novel approach to epidemiological feature detection, which is outlined in Fig. 1. Prior to analysis, all data was smoothed in MATLAB using a gaussian-weighted moving average filter with a window length of 25 days to obtain quasi-continuous trends, as shown in Fig. 1A, which were more suitable for peak deconvolution than the noisy raw data. Pairs of corresponding case-death trends for each region of interest were min–max normalized between 0 and 1 to maintain consistency in our analyses across these regions.

The regions and waves of interest we analyzed were mainly selected for the purposes of demonstrating this novel approach and follow a set of general selection criteria. Regions of interest with consistent, daily reporting of cases and deaths, which also showed distinct surges, were chosen as they facilitated data smoothing and peak deconvolution. Higher income countries reporting peak surges upwards of 5000 cases per day generally fit these criteria often due to the greater capacities of their healthcare networks to document disease progression.

While Israel, Canada, and Germany all meet the general selection criteria, they were also selected based on their unique administrative structures and public health responses during their respective second waves of the pandemic. These three countries exhibit a broad spectrum of administrative centralization and decision-making agility at national and subnational levels. Israel has a unified centralized administration whereas Canada’s administration is more decentralized with voluntary collaboration at a national level, and that of Germany is the most decentralized yet abided by consensus-based decision making at the federal level during their second wave of COVID-19. These three distinct scenarios provided a basis for exploring how the presented methods could be used to study the effects of COVID-19 management on CDL and aCFR during similar periods of COVID-19 progression.

The second wave of the pandemic in each region of interest was selected for analysis based on the assumption that COVID-19 cases and deaths were more accurately reported following the first wave due to constant improvements in testing and reporting infrastructure through the early stages of the pandemic. Additionally, the second wave was chosen instead of later waves to investigate COVID-19 disease landscapes prior to widespread vaccine rollouts. Although these selection criteria were used to guide the present analysis, this approach to epidemiological feature detection is broadly applicable to the study of disease landscapes beyond nationally reported surges in COVID-19 cases and deaths, including regions with sparser disease reporting.

### Peak deconvolution

Peak deconvolution was used to deconstruct national time series trends from the second wave of the COVID-19 pandemic into their component peaks, or sub-waves, as shown in Fig. 1B. Following data selection and processing in MATLAB, peak deconvolution was performed using Fityk, an open source curve fitting and data analysis software^25^. The quality of the fit for each of the deconvolved time series trends was assessed based on their coefficient of determination, R^2^, where an R^2^ value greater than 0.99 was considered acceptable for this analysis.

To fit corresponding case-death surges within a region of interest, time windows of the same length for each data type were offset to account for the average CDL between trends. The offset between case and death time windows was defined by maximizing the cross-correlation function between case-death surges. Although time windows were constrained to be the same length for the case and death trends within each region of interest, they were allowed to vary between regions as the duration of the second wave was unique to each region studied.

For the purposes of peak deconvolution, the time window ultimately defined the distance between the center of the first and last deconvolved peaks flanking case-death surges, which, for example, is indicated in Fig. 1B as *t*_*window*_ for the trend in daily deaths. Each trend can be fit with at least 3 peaks (i.e., two flanking peaks and 1 unconstrained peak), and the number of peaks can be increased to enhance the resolution of feature extraction. Corresponding case-death trends within a region of interest must be fit with the same number of peaks to ensure correspondence between individual features extracted from the case and death landscapes. For instance, Fig. 1B shows a pair of case-death trends fit with 6 peaks each, of which the 4 central peaks from each trend are shown as isolated peak pairs in Fig. 1C.

Each of the trends in this paper were deconvolved into 6 peaks, which proved to be the minimum number of peaks that accurately fit all these trends (i.e., the number of peaks was limited by the most feature-rich landscapes). While it is possible to fit these trends with greater numbers of peaks representative of increasingly finite contributions to these disease landscapes, the minimum number of peaks was chosen to simplify the present analysis, reduce the likelihood of overfitting, and study the broadest population-level insights that may have tangible meaning at a national level. Moreover, consistent peak fitting across each region of interest provided uniformity of model outputs, which facilitated comparisons of interregional disease progression.

Each isolated peak pair comprises a case and death peak of identical widths and represents a unique epidemiological feature. Peak widths may vary across isolated peak pairs but are kept constant between the case and death peaks of each individual pair to consolidate the temporal parameterization of each feature. CDL is defined as the center-to-center distance between the case peak and death peak of each isolated peak pair, which is indicated as *∆* in each of the subplots in Fig. 1C. The peak deconvolution process yields a sequence of distinct and localized features from real-world data that can be further parameterized with compartmental models of disease transmission.

### SIRD modelling

The SIRD model, shown in Fig. 1D, is a variation of the susceptible-infected-recovered (SIR) model which explicitly accounts for disease-induced deaths among infected populations. In the SIRD model, infected individuals either recover from disease with natural immunity or die due to infection. The progression of the disease landscape is represented by a set of four ordinary differential equations which each describe the dynamics of a different SIRD compartment, as follows:

1 $$\frac{dS}{{dt}} = - \beta IS,\;\frac{dI}{{dt}} = BIS - \gamma I - \mu I,\;\frac{dR}{{dt}} = \gamma I,\;\frac{dD}{{dt}} = \mu I$$

here *β* is the transmission rate constant, *γ* is the recovery rate constant, and *μ* is the mortality rate constant. Unlike the SIR model, the SIRD model includes an explicit analytical solution for the deceased population, *D*, which enables simultaneous modelling of death data in addition to case data. Cases and deaths are among the most commonly reported epidemiological data worldwide and serve as the basis for our CDL analyses.

The SIRD model does not explicitly consider many of the complexities surrounding disease progression, such as incubation periods, asymptomatic transmission rates, and restrictive public health measures. Additional model compartments, such as an exposed (E) compartment used in SEIRD models of COVID-19 dynamics, are omitted from this analysis for simplicity. In many cases, simpler models have been shown to be sufficient for modelling of COVID-19 trends on timescales of several days to a couple months^26^ and observationally equivalent to their more complex counterparts in these situations^2,27^.

Vital dynamics, which include births and natural deaths, are not considered in the SIRD model. These processes are generally used for modelling endemic diseases, which persist in populations over longer periods of time upwards of a decade^28^. Here, we assume that natural deaths are negligible compared to those caused by disease and similarly assume that birth processes are insignificant to overall population dynamics at the timescales presented.

We also assume that natural immunity is conferred to recovered individuals at least temporarily such that they are not reintroduced into the susceptible population following recovery. Immune memory has been shown to persist for upwards of 3 months in most individuals following COVID-19 infection^29,30^ with little evidence of reinfection within similar timeframes^31^. In our analysis, each pair of case-death peak fits are simulated with a unique SIRD model and typically span between 10 and 15 days of the 105–120 days over which most second waves are observed. Given these considerations, the simplifying assumptions of the SIRD model without vital dynamics are reasonable for the timescales being studied.

While the case and death data for peak deconvolution are shown as the change in daily counts (i.e., new cases and deaths), the explicit analytical solutions of the SIRD model are expressed as total daily counts as shown in Figs. 1E1 and 2. To convert the population dynamics of the model to daily changes in the SIRD populations, we take the numeric derivative of the model’s analytical solutions using the forward difference formula, which are shown in Figs. 1F1 and 2. New daily cases are approximated using the negative time derivative of the susceptible population, *S*, given that changes in *S* are governed by a single term as shown in Eq. (1), which describes the rate of irreversible transition between susceptible and infected populations. Analogously, the time derivative of the deceased population, *D*, is used to approximate new daily deaths.

**Figure 2:**
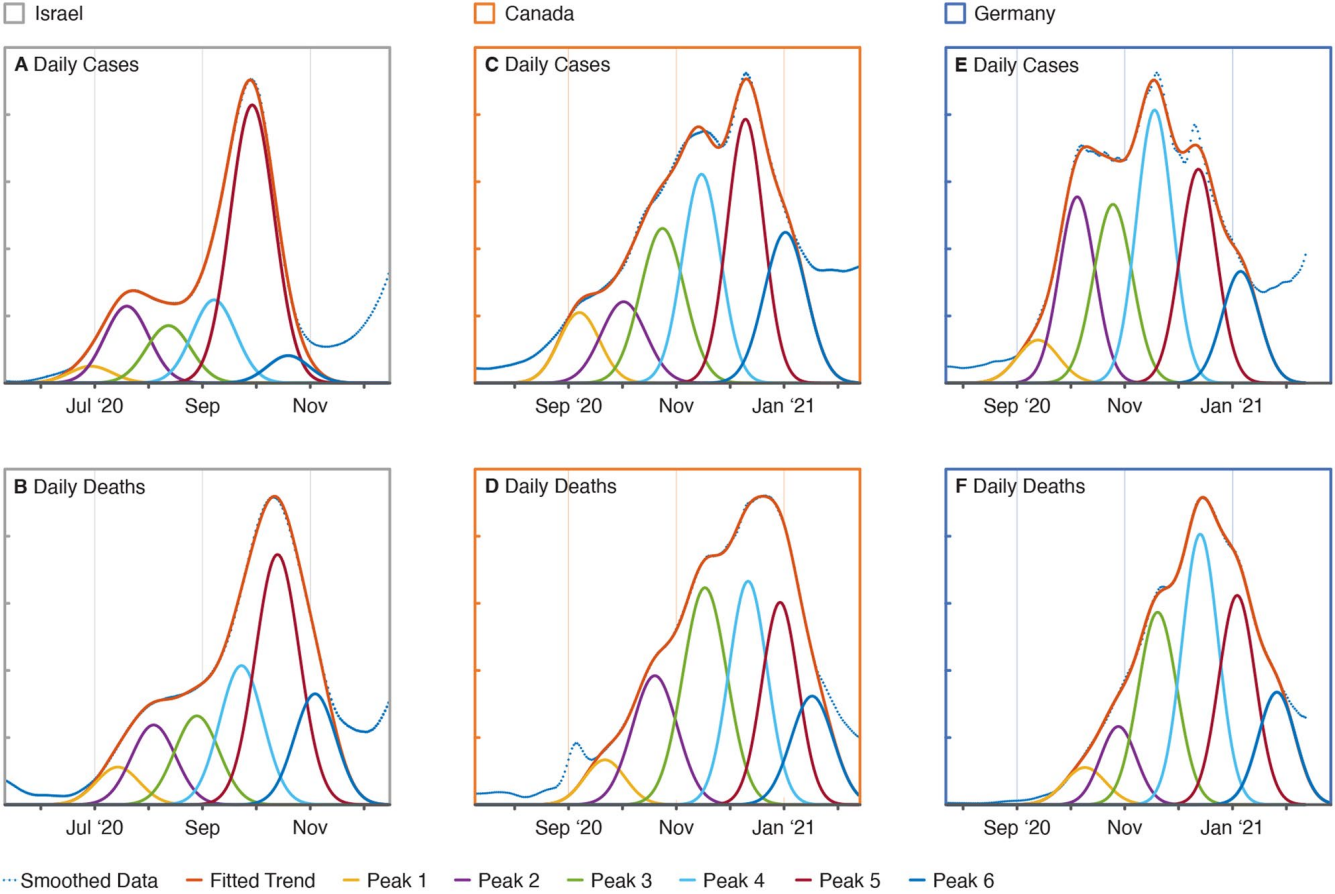
Peak fits for national daily cases and deaths reported from the second wave of the COVID-19 pandemic: (**A**) Israel daily cases, (**B**) Israel daily deaths, (**C**) Canada daily cases, (**D**) Canada daily deaths, (**E**) Germany daily cases, and (**F**) Germany daily deaths.

Modelled cases and deaths are fit to pairs of deconvolved case-death peaks using non-linear least squares regression, which is illustrated in Fig. 1G. The optimization process works to minimize the sum of squares error between the model and reported data over the time interval spanning the case-death peak pair of interest. In Fig. 1G, the error is considered minimized and model parameters optimized when the parameterized modelled peaks (yellow and red dashed lines) and their respective deconvolved peaks (in blue) are practically superimposed. Minimization of the model error is defined by the objective function, *f(x)*, as follows:

2 $$min\|f\left( x \right)\|^{2} = min\left[ {\left( {C_{d} - C_{m} \left( x \right)} \right)^{2} + \lambda \left( {D_{d} - D_{m} \left( x \right)} \right)^{2} } \right]$$

where *C* and *D* represent case and death peaks, respectively, and are denoted with a *d* for deconvolved peaks or *m* for SIRD-modelled peaks. Using mathematical notation, Eq. (2) describes how model error is calculated during each iteration of the parameter optimization process. In the objective function, *f(x)*, *x* is a vector variable of the parameters and initial conditions used in the SIRD model and *λ* is a scaling factor intended to compensate for the general disparity in the magnitude of cases compared to deaths. Model parameters include the transmission, recovery, and mortality rate constants while initial conditions include the initial susceptible, infected, and recovered populations.

For simplicity, we constrained initial recovered populations to zero and kept initial infected populations arbitrarily low compared to their corresponding initial susceptible populations (i.e., a ratio of approximately 1:100,000 infected to susceptible individuals). The initial susceptible population of each peak pair model was scaled proportionally to the relative area occupied by its death peak as mortality was assumed to be a more reliable indicator of disease prevalence than cases due to variability in testing rates within and across regions of interest throughout the pandemic^32^.

Each pair of modelled case-death peaks represents a unique SIRD simulation generated from a single set of optimized parameter values (i.e., *β*, *γ*, and *μ*), which ensures that corresponding case-death data for each isolated peak pair is satisfied under the same epidemiological conditions. Model parameter values of different peak pairs are independent of one another. During the simulation process, modelled peaks are also temporally shifted to be aligned with the time series data and ultimately result in a purely mathematical recreation of the original time series trends as illustrated in Fig. 1H.

## Results

### Peak analysis

Nationally reported data from the second waves of the COVID-19 pandemic in Israel, Canada, and Germany were analyzed using the presented peak deconvolution and SIRD modelling methodology to investigate the relationship between CDL and other epidemiological parameters shaping these disease landscapes. Daily case and death trends for each country were fit with 6 peaks as shown in Fig. 2. While the scale of the x-axes is the same for each of the subplots, Israel’s second wave started approximately 100 days before those of Canada and Germany.

The durations of the second waves of cases and deaths, which were calculated as the number of days between the centers of the two peaks flanking each fit (i.e., peaks 1 and 6), were between 110 and 115 days for all three countries. The consistency of the second wave durations across these countries is suggestive of an epidemiologically invariant phenomenon underlying these transient surges in the spread of COVID-19. Despite this similarity, the deconvolved case and death trends shown in Fig. 2 depict a unique disease landscape for each country within each of their second waves. The surge in Israel’s daily cases shown in Fig. 2A has a backloaded bimodal distribution, whereas Canada’s cases (Fig. 2C) increase steadily; both of which are distinct from Germany’s sudden and relatively sustained spike in daily cases (Fig. 2E). As expected, surges in daily deaths shown in Fig. 2B,D,F lag behind their respective surges in daily cases and also bare shaped-based resemblances to them.

The similarity between the respective trends in second wave cases and deaths was confirmed and compared by calculating the Pearson correlation coefficients and dynamic time warping (DTW) minimum distances for each of the pairs of case-death time series shown in Table 1. These similarity measures were calculated using independently normalized case and death trends, which were temporally shifted to maximize their cross-correlation functions. The Pearson correlation coefficients for each country are greater than 0.95, which indicates a strongly positive correlation between the national case and death trends of each country. The similarity between respective trends confirms a level of consistency between the reporting of case and death data within each of these countries required to fit and model the same number of analogous case-death peaks in the analyses presented. Additionally, the Pearson correlation coefficients and DTW minimum distances both show that Canada’s case-death trends are most similar, followed by those of Israel and then Germany.

**Table 1:** Correlation between the deconvoluted trends in daily cases and deaths in Israel, Canada, and Germany during the second wave of the COVID-19 pandemic.

Country	Pearson correlation coefficient	DTW minimum distance
Israel	0.977	1.332
Canada	0.991	1.173
Germany	0.961	1.946

### Model validation

**Figure 3:**
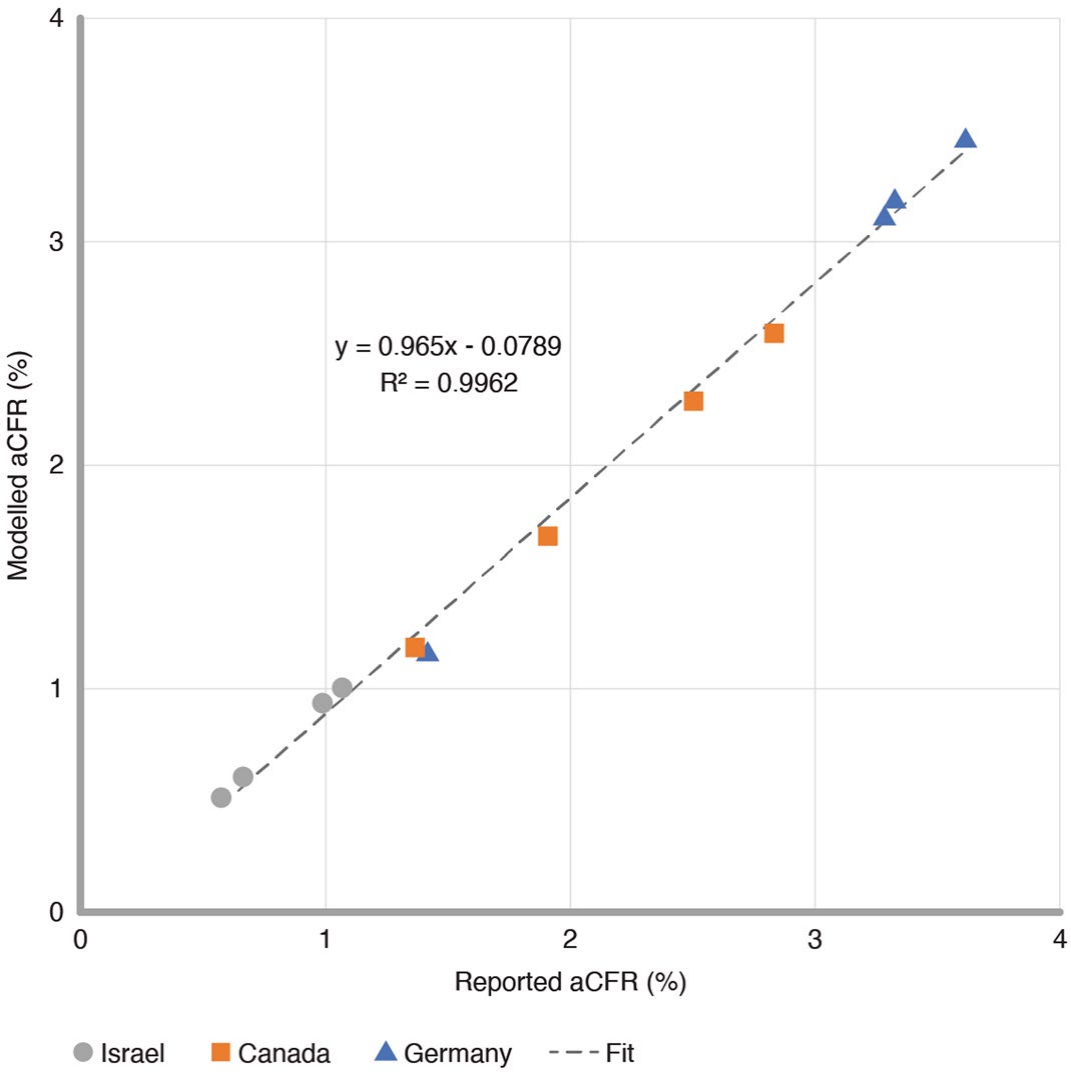
Reported peak-by-peak aCFRs compared to aCFRs calculated from SIRD model parameters in Israel, Canada, and Germany during the second wave of the COVID-19 pandemic.

To validate the SIRD model, we first calculated and compared the adjusted case fatality rate (aCFR) for each of the deconvolved and modelled peak pairs. CFR is defined as the ratio of deaths to cases over a specified period of time while aCFR accounts for CDL and is defined as the ratio of deaths to cases where the deaths are temporally offset by their respective latency. This epidemiological measure is generally reported as a percentage, where larger percentages are indicative of more severe disease outcomes. The majority of nationally reported CFRs for COVID-19 based on aggregate data range between 0.5–5.0%: Israel reporting 0.6%, Canada 1.7%, and Germany 2.2%, as of October 2021. For the deconvolved peak pairs, aCFRs were calculated by dividing the area under each death peak by that of its corresponding case peak. The corresponding rates for each modelled peak pair were similarly calculated using their respective SIRD model parameters as follows:

3 $$Modelled\;{\text{a}}CFR = \frac{\mu I}{{\beta IS}} \approx \frac{\mu }{{\beta S_{0} }}\quad where\;I \ll S$$

here in addition to the rate constants, *β*, *I*, and *μ*, introduced in Eq. (1), *S*_0_ is the initial susceptible population for each modelled peak pair. Figure 3 provides a comparison of the reported and modelled aCFRs within and between each country. As expected, the plot shows a strong linear correlation (R^2^ greater than 0.99) with an equal proportionality (slope of 1.0), which demonstrates that the aCFRs calculated for each of the isolated peak pairs are closely and consistently approximated by their respective SIRD model parameters. Figure 3 also shows a clear separation between the reported second wave disease landscapes of each of these countries, where Israel has the lowest aCFRs on average, followed by Canada, and then Germany. Moreover, the variation in aCFRs between peak pairs from the same country highlights the potential for peak deconvolution methodologies to identify increasingly granular features of these disease landscapes.

### Case-death latency

**Figure 4:**
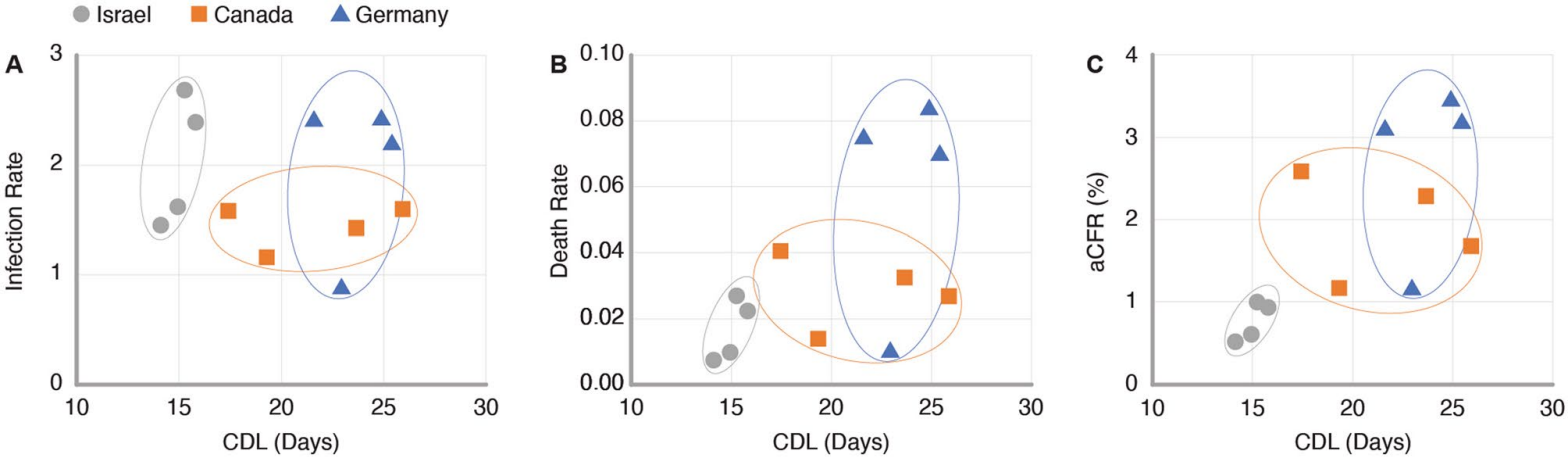
CDL compared to modelled infection rates (**A**), death rates (**B**), and aCFRs (**C**), in Israel, Canada, and Germany during the second wave of the COVID-19 pandemic.

While aCFRs provide an overview of these disease landscapes, studying their underlying infection and death rates enables a deeper understanding of the epidemiological parameters most heavily influencing each country’s pandemic response. Figure 4 shows the modeled infection, death, and aCFRs versus CDLs for each of the isolated peak pairs for Israel, Canada, and Germany. The plot of the recovery rates versus latencies is not shown as it closely resembled that of the infection rates shown in Fig. 4A, given that more than 95% of reported cases led to non-fatal disease outcomes across all three countries. In Fig. 4, peak pairs—each represented by an individual data point—are uniquely defined within each plot and clustered by country, which illustrates the dynamic variability of these disease landscapes even within a single wave of the COVID-19 pandemic. In Germany, such time dependent variations in aCFRs during the second wave outbreak have been linked to changes in the age distribution of confirmed cases^33^.

Between countries, death rates (Fig. 4B) varied significantly more than infection rates (Fig. 4A). Based on the weighted average rates for each country reported in Table 2, national death rates showed a 76% relative standard deviation (RSD), whereas infection rates, which showed an 18% RSD, were relatively constant. These observations indicate that death rates disproportionately impacted the magnitude of aCFRs compared to infection rates during the second wave of the COVID-19 pandemic in Israel, Canada, and Germany. They also suggest that the risk of COVID-19 infection was relatively independent of country and time considering that all three countries reported similar testing rates per capita during their respective second waves with an average of 2.8 ± 0.6 daily new tests per 1000 people^34^.

**Table 2:** Weighted averages of CDLs, infection rates, death rates, and aCFRs calculated from peak fits and SIRD model parameters in Israel, Canada, and Germany during the second wave of the COVID-19 pandemic.

Country	CDL (days)	Infection rate	Death rate	aCFR (%)
Israel	14.631	1.791	0.013	0.661
Canada	21.997	1.404	0.026	1.776
Germany	23.827	2.024	0.062	2.801

Studying case death latency provided further segmentation of these epidemiological parameters by country. For the three countries presented, we observed that the average death rates and aCFRs were directly proportional to CDL. In particular, the average aCFRs showed a strong linear correlation (R^2^ = 0.91) to the latencies reported in Table 2, which is also illustrated in Fig. 4C. Overall, this trend is suggestive of a more complex relationship between disease severity and the temporal dynamics of disease progression and reporting both at an individual and societal level, which needs to be considered when comparing aCFRs across multiple countries.

## Discussion

To further investigate the strongly positive correlation between CDL and aCFR across Israel, Canada, and Germany during the second wave of the COVID-19 pandemic, multiple underlying factors must be considered. One of the main factors governing the trends in the disease landscapes across these three countries are the governments themselves^35^. The governments of Israel, Canada, and Germany each embody unique administrative structures, which influence the unity of their pandemic responses at local, state, and federal levels. Based on our analyses of CDL and aCFR, we consider how governments with increasingly decentralized and codependent administrative structures may be more likely to experience worse disease outcomes due to a lack of decisive policy making and timely access to systematic healthcare data.

The role of government in pandemic preparedness and responsiveness has been shown to impact disease landscapes; for example, with government effectiveness being negatively associated with COVID-19 mortality^36^. Although government effectiveness is quantified as a Worldwide Governance Indicator (WGI)^37^, it considers the quality of government services beyond those related to public health and is based on aggregate data reported on a yearly basis. These limitations make it difficult to evaluate the impact of government interventions and countermeasures at critical transient moments throughout the COVID-19 pandemic (e.g., surges in cases and deaths).

While the true degree of government effectiveness in each of these countries is difficult to quantify, broad comparisons of their different administrative structures and subsequent pandemic responses—informed by factors including population size, distribution, and segmentation—provide insights into the correlation across countries between CDLs and aCFRs. For example, Israel has a population of 9 million people living across 6 districts within a unitary state of centralized federal governance. Under this government, laws and public health policies are exclusively implemented at the federal level (e.g., countrywide lockdowns). During the second wave of the pandemic, Israel opted to decentralize the management of COVID-19 to the country’s four universal health plans, which oversee the administration of primary care services such as testing and patient education^38^, and municipalities had the option to implement additional health measures based on a classification system of local disease severity set forth by Israel’s Ministry of Health^39^. However, these health plans ultimately belong to a system of direct oversight by the State, which resulted in a nationally homogeneous second wave pandemic response and lower CDLs as well as aCFRs in Israel compared to those of Canada and Germany.

Canada and Germany are both governed as federations, which operate on a spectrum of shared power distributed between state and federal levels. In Canada, which has a population of 38 million people living across 10 provinces and 3 territories, neither provincial governance nor federal jurisdiction are subordinate to the other. Instead, Canada’s provincial and federal governments act autonomously to exercise their respective constitutional responsibilities and only coordinate policies through voluntary negotiations where there is mutual interest in intergovernmental collaboration^40^. Based on this system of governance, the early stages of the pandemic in Canada saw mask mandates and regional lockdowns implemented at the provincial level while the federal government maintained national border closures as well as international travel restrictions including mandatory quarantining for return travellers^41,42^. Canada’s heterogeneous second wave pandemic response, which was highlighted by provincial autonomy and cooperative national decision making, led to moderate aCFRs with the largest variation in CDLs compared to Israel and Germany.

Germany, which has a population of 83 million people living across 16 states (known as Länder), responded in much the same way as Canada with the main differences arising from Germany’s consensus-based federal system. With lawmaking abilities predominantly resting in the states’ hands and requiring unanimity from state leaders^40^, Germany’s second wave pandemic response was guided by fragmented governance and fractious federal-state relations. Despite the implementation of delayed and compromising nationwide lockdowns, rules around local social restrictions, face masks, and other public health policies varied across each Länder^43^. The decentralized healthcare systems of Germany’s states have presented a heterogeneous response throughout the pandemic, which has ultimately resulted in substantial differences in disease landscapes across the country, especially during the second wave of the pandemic^44^. Of the three countries presented, Germany showed the largest CDLs and aCFRs during their second wave. However, the range of CDLs in Germany’s second wave (21.6–25.4 days) was smaller than that of Canada’s (17.4–25.9 days), which may reflect Germany’s more unified response across Länder.

Decentralized governments, such as the federal systems in Canada and Germany, tend to exhibit patchwork responses to large scale public health outbreaks^45^. Non-standardized disease reporting across these heterogeneous policy and data collection landscapes often leads to a lack of timely and systematic healthcare data, which can hamper pandemic responsiveness^43^. In Germany, for example, delays in registered COVID-19 deaths (i.e., from the date of death to date of publication) of one to three weeks were common throughout the second wave of the pandemic^19^. Overall, the decentralization of COVID-19 management may be a contributing factor to the higher CDLs and aCFRs observed during the second waves of the pandemic in Canada and Germany compared to Israel.

For Germany, the codependency of state governments to enact policies at a national level may have also contributed to the country having the highest CDLs and aCFRs of the three countries analyzed. However, timely pandemic responses are important to smaller unified governments. For example, Israel’s more severe second wave has been attributed to delayed government action during the early weeks of the country’s second wave outbreak^46^. Ultimately, unified governments and healthcare networks are more likely to effectively respond to emerging outbreaks and report on them in a way that reflects their true disease landscapes, whereas decentralized networks experience greater latencies, which are more prone to underestimates in disease severity and costly delays in implementing public health interventions and countermeasures.

While administration and governance are overarching factors which have undoubtedly contributed to the progression of the pandemic, CDLs and aCFRs are also influenced by other confounding factors, especially at subnational levels. Such factors may include the emergence of different variants of concern throughout the second wave of the pandemic, which are associated with a higher risk of mortality compared to earlier variants for cases running longer clinical courses (i.e., more than 2 weeks since diagnosis)^47^. Population level factors such as age demographics are also important to consider, as higher case fatality rates are disproportionately observed among older populations^48^ while the relationship between age and CDL is relatively unexplored^49^. Other socioeconomic factors such as access to healthcare resources and GDP per capita also play a significant role in shaping these disease landscapes^50^. These additional factors and determinants of disease are areas of interest for future work, especially as relevant stratified data for each of these considerations continues to become more available.

As the pandemic progresses, new types of data also become available, allowing for further analyses of emerging waves. The methodologies introduced in this paper could be adapted to analyze subsequent waves of COVID-19 by updating the SIRD model to account for new types of data representative of additional model compartments. As an example, analyses of third wave data could provide unique insights on the early impacts of vaccinations, which could be studied alongside cases and deaths by adding model compartments that account for vaccinal and waning immunities^3^. Apart from updating the SIRD model to reflect relevant data and disease dynamics, the presented approach of epidemiological feature detection is broadly applicable to any disease landscape for which there is sufficient time series data to deconstruct into constituent peaks.

Furthermore, analyzing additional regions at various scales would allow for more conclusive findings to be drawn regarding the methodology presented here. Analyzing and comparing smaller regions, such as cities, provinces, or states, could provide more detailed insights into the spatiotemporal characteristics of disease progression, such as when individuals travel from one homogenous region to another. These analyses could additionally be used to explore temporal features of disease landscapes previously studied using mobility networks and metapopulation approaches^51,52^. Ultimately, the versatility of the epidemiological feature detection method presented here makes it applicable to a wide variety of applications within epidemiology.

## Conclusions

In this paper, we introduced and applied a novel latent mixture approach, which we coin epidemiological feature detection, to analyze the second wave of the COVID-19 pandemic in Israel, Canada, and Germany. Applying this approach, we used peak deconvolution methods to extract and relate distinct and localized features from trends in daily cases and deaths, and we further characterized these features using a SIRD model of disease transmission to quantify spatiotemporal variations in epidemiological parameters including infection, death, and recovery rates. We found that the average death rate across all three countries varied more than 4 times as much compared to the average infection rate, which suggests that higher death rates, as opposed to lower infection rates, are the main drivers of increases in adjusted case fatality rates. Additionally, we found a strongly positive correlation (R^2^ = 0.91) between average adjusted case fatality rate and the lag time between reported cases and deaths of isolated features, which we term case-death latency. Of the three countries presented, Israel showed the lowest average case-death latency and adjusted case fatality rate (14.6 days and 0.7%), followed by Canada (22.0 days and 1.8%), and Germany (23.8 days and 2.8%). We further discuss this trend in the context of increasingly decentralized governments and administrative structures in these respective countries. We highlight the importance of cooperative decision making and timely access to systematic healthcare data for effective responses to emerging public health outbreaks. Overall, this work emphasizes the need for new empirical approaches to complement ongoing pandemic scenario planning initiatives and illustrates the potential for epidemiological feature detection to improve health security and pandemics preparedness for COVID-19 and beyond.

## Data Availability

The COVID-19 case and death data used in this paper can be downloaded from the John Hopkins Coronavirus Resource Center’s (CRC) open access database. The code and data used to execute our novel approach to epidemiological feature detection, which is outlined in Fig. 1 and shown in Figs. 3 and 4, will be available for download from GitHub. Peak fits shown in Fig. 2 were generated using Fityk, an open-source curve fitting and data analysis software and will be available for download from GitHub.
